# Notch1/2/3/4 are prognostic biomarker and correlated with immune infiltrates in gastric cancer

**DOI:** 10.18632/aging.102764

**Published:** 2020-02-06

**Authors:** Jian Hu, Jianghong Yu, Jun Gan, Ning Song, Liubin Shi, Jie Liu, Ziqiang Zhang, Jianjun Du

**Affiliations:** 1 Department of General Surgery, Huashan Hospital, Fudan University, Shanghai 200040, China; 2 Department of Digestive Diseases of Huashan Hospital and Institute of Biomedical Sciences, Fudan University, Shanghai 200040, China; 3 Department of General Surgery, Huashan Hospital North, Fudan University, Shanghai 200040, China

**Keywords:** Notch, gastric cancer, prognosis, immune infiltrates

## Abstract

Notch refers to a set of genes encoding a family of transmembrane receptors in mammalian cells. Previous evidence indicated that Notch receptors were implicated in the onset of gastric cancer. However, there is little evidence on the different genetic expression patterns of the four Notch receptors and their values for patient prognosis. Most recently, we investigated the transcriptional data of Notch receptors and related patient survival in patients with GC based on several databases, including ONCOMINE, GEPIA, Kaplan–Meier Plotter, cBioPortal and TIMER. According to our findings, gastric cancer tissues, compared with adjacent normal tissues presented a higher level of expression of Notch1/2/3. We also performed a survival analysis and noted that gastric cancer patients with high transcription levels of Notch1/2/3/4 had a low relapse-free survival. In gastric cancer patients, higher levels of infiltration in their CD4+ T cells, macrophages, neutrophils, and dendritic cells were positive associated with the expression of Notch receptors. Notch expression had significant association with diverse immune marker sets in gastric cancer. Overall, this study provides evidence that Notch1/2/3/4 could become the potential targets for precision treatment and new biomarkers in the prognosis of gastric cancer.

## INTRODUCTION

Gastric cancer (GC) is a very common disease worldwide and has the second highest mortality rate among all cancers. In the past decade, researchers have found that the deregulated expression of specific genes can increase the risk of GC. According to microarray analysis, previous studies revealed that in GC tissues the expression of specific genes is different from that in adjacent normal tissue. Although significant progress has been achieved in GC diagnosis and treatment, the five-year survival of patients is still unsatisfactory [1]. Researchers have identified epigenetic and genetic alterations as some of the main factors inducing GC. However, the underlying molecular pathogenic mechanisms on molecular level are still obscure. Hence, it is important to identify prognostic markers and potential drug targets to enhance prognosis and individualized treatments.

The Notch signaling pathway is highly conserved among various species. In mammals, four type I transmembrane Notch receptors (Notch 1-4) are synthesized, all of them with unique roles during the generation of immunocytes [2, 3]. Notch signaling also exerts important function in the development and tissue homeostasis of various organ [4, 5]. Provided the importance of Notch signaling in regulating cellular behavior, it is perhaps not surprising that Notch also has an important role in many types of cancer, particularly due to its importance in the regulation of stem and progenitor cells. Several mechanisms such as epigenetic regulation, posttranslational, modifications, gene overexpression and mutations, may lead to the dysregulation of the Notch pathway [2]. Interestingly, Notch activity is associated with oncogenic and tumor-suppressive functions [6, 7]. It is involved in cell survival, cell death pathways, proliferation and growth arrest, as well as cell differentiation into terminally differentiated cells versus cancer cell “stemness” [8]. These functions provide evidence of a context-dependent nature of Notch-induced cellular reactions.

The onset of GC can be described as the result of interactions between a series of factors concerning genetics, epigenetics and the external environment, which jointly lead to the deregulation of the signaling pathways that may induce the onset of cancer [9, 10]. Further, there has been a general belief that it is the dysfunctional oncogenic pathways that induce the onset of GC, which may include the epidermal growth factor receptor (EGFR), Notch, Hedgehog, nuclear factor-κB and Wnt/β-catenin pathway [11]. Among these pathways, Notch signaling is involved in direct cell-cell communication, cell differentiation, proliferation and apoptosis [12].

The balance between immune effector cells in the tumor microenvironment helps the malignant cells escape from the immune response. Tumor infiltrations of tumor associated macrophages, neutrophils, regulatory T cells are correlated with poor prognosis [13–15]. Tumor infiltrating CD8+ cytotoxic T lymphocytes and DCs are generally associated with favorable outcome of GC [16, 17], although some subsets of these immune cells have inverse prognosis prediction values. High ratios of Foxp3+/CD4+ and Foxp3+/CD8+ in tumors are associated with a poor prognosis [18, 19]; whereas high Th1/Th2 ratio in tumors predicts a good prognosis [20].

In previous studies, researchers have investigated the consequences of dysregulation of the Notch pathway and how it relates to clinicopathological features and prognosis in human GC. Nevertheless, the role of Notch family members in the development and progression of GC remains unknown. This study aims to address this question through in-depth analysis of the mutational activation and expression of Notch family members and their link with prognosis and immune infiltrates in GC patients.

## RESULTS

### Transcriptional levels of Notch in patients with GC

**Figure 1:**
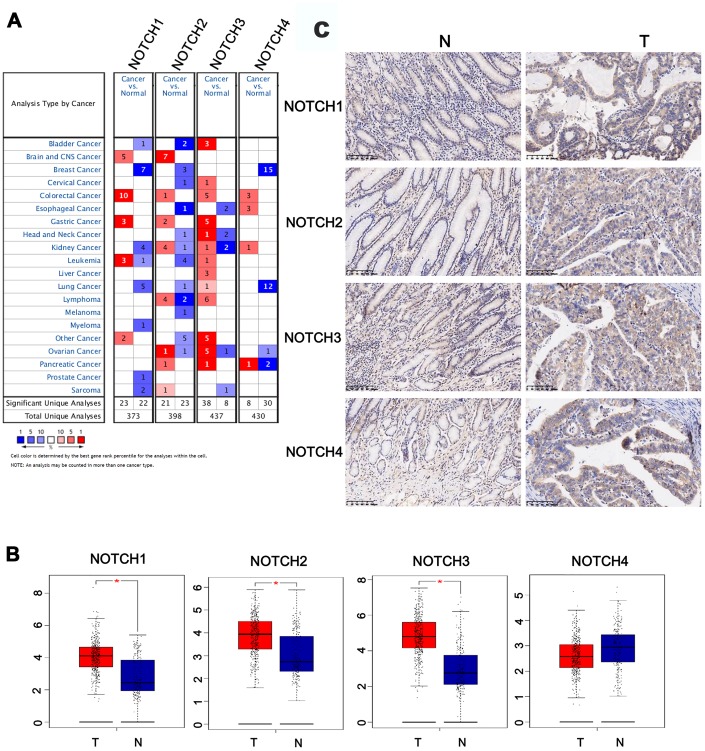
**The expression of Notch in different tissues.** (**A**) The transcription levels of Notch receptors in different types of cancers. (**B**) The expression of Notch receptors in GC. (**C**) The expression of Notch protein in GC. Students’t-test was used in comparative analysis of Notch expression. T: tumor, N: normal tissue, number (T) = 415, number (N) = 211.

Using the ONCOMINE databases, a comparative analysis investigating transcription levels of Notch receptors was performed on cancer tissues and adjacent normal tissues (Figure 1A). According to the information from five datasets, a significant upregulation of Notch3 mRNA expression was detected in GC patients. In Chen’s dataset [21], the expression of Notch3 in gastric adenocarcinoma was 1.594 and 1.871 times respectively of that in the samples of normal tissue (Table 1). In Wang’s dataset [5], the expression of Notch3 in GC tissue was 2.549 times of that in normal tissue. In DErrico’s dataset [22] the expression of Notch3 in gastric intestinal type adenocarcinoma was 2.869 times of that in normal tissues. In Cho’s dataset [23] the expression of Notch3 in GC tissue was 1.630 times of that in normal tissue. Chen [21] showed that the expression of Notch1 was also higher in cancer tissues, since the expression in gastric intestinal type adenocarcinoma and diffuse gastric adenocarcinoma was 1.920 and 1.733 times respectively of that in patients with normal gastric tissue, respectively (Table 1). Based on the dataset of DErrico, the expression of Notch1 in gastric intestinal type adenocarcinoma was also 2.247 times of that in normal tissue [22]. In comparison with normal tissue, GC exhibited 1.625 [5] and 1.832 [22] times expression levels of Notch2, respectively.

**Table 1:** The significant changes of Notch expression in mRNA level between different types of GC and stomach tissues.

	**Type of GC VS. Stomach**	**Fold Change**	**P value**	***t*-test**	**Ref**
NOTCH1	Gastric intestinal type adenocarcinoma vs. Normal	2.247	1.04E-13	9.844	DErrico [14]
	Diffuse gastric adenocarcinoma vs. Normal	1.733	6.14E-8	6.700	Chen [13]
	Gastric intestinal type adenocarcinoma vs. Normal	1.920	9.17E-11	7.739	Chen [13]
NOTCH2	Gastric cancer vs. Normal	1.625	5.76E-5	5.128	Wang [5]
	Gastric intestinal type adenocarcinoma vs. Normal	1.832	3.15E-8	6.617	DErrico [14]
NOTCH3	Gastric cancer vs. Normal	2.549	1.42E-6	6.232	Wang [5]
	Diffuse gastric adenocarcinoma vs. Normal	1.871	4.15E-8	7.496	Chen [13]
	Gastric intestinal type adenocarcinoma vs. Normal	1.594	4.15E-10	7.175	Chen [13]
	Gastric intestinal type adenocarcinoma vs. Normal	2.869	1.14E-9	7.723	DErrico [14]
	Diffuse gastric adenocarcinoma vs. Normal	1.630	7.15E-5	4.142	Cho [15]
NOTCH4	NA	NA	NA	NA	NA

Ref: reference

### Expression levels of Notch in GC patients

The comparative investigation was conducted between the Notch mRNA expression in normal and gastric adenocarcinoma tissues based on the information provided by the GEPIA (Gene Expression Profiling Interactive Analysis) dataset. Based on the findings, in comparison with normal gastric tissues, gastric adenocarcinoma tissues presented higher expression levels of Notch1/2/3 (Figure 1B). The expression of Notch1, Notch2, Notch3 and Notch4 proteins, which were examined by immunohistochemistry, were higher in the gastric adenocarcinoma tissues than that in the normal gastric tissues (Figure 1C).

### Association of Notch1/2/3/4 mRNA expression with the prognosis of GC patients

**Figure 2:**
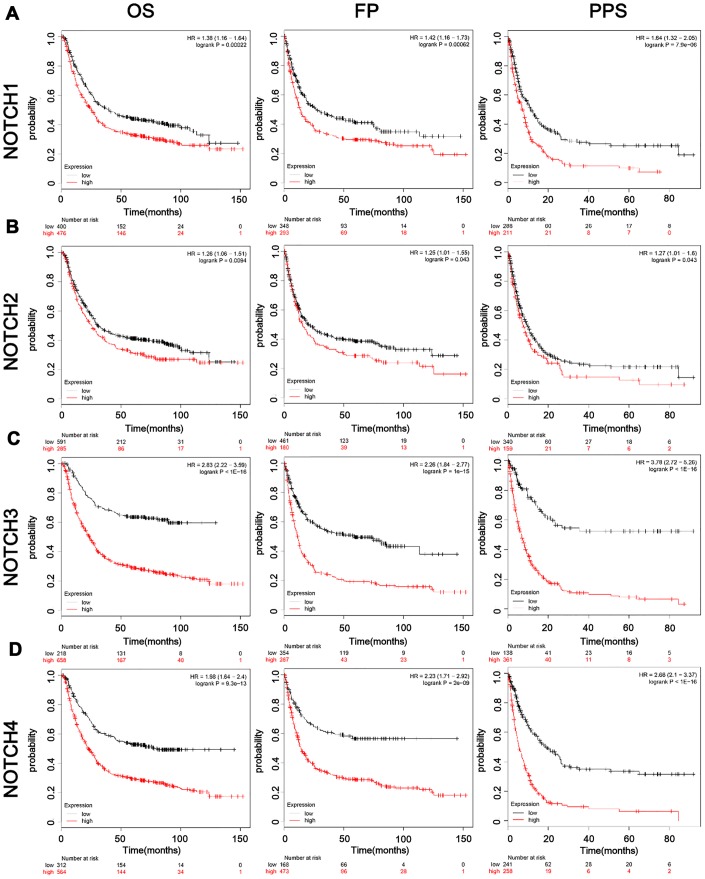
**The prognostic value of mRNA level of Notch receptors in GC patients.** (**A**–**D**). Logrank test was used in analysis of OS/FP/PPS.

**Figure 3:**
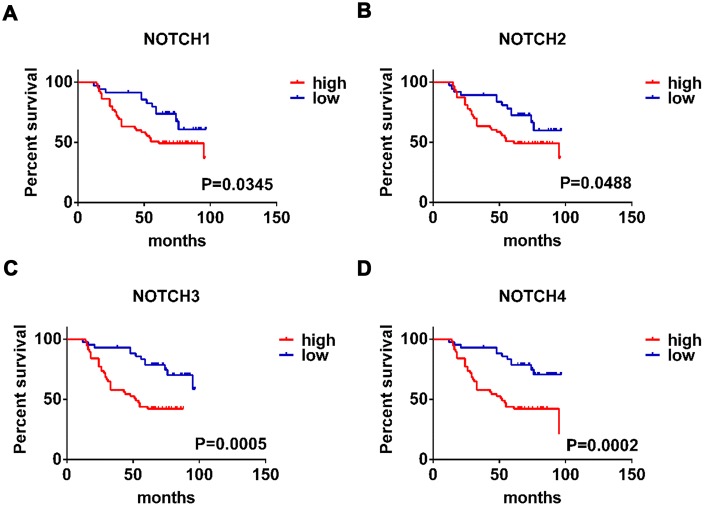
**The prognostic value of expression level of Notch receptors in gastric adenocarcinoma patients.** (**A**–**D**). Logrank test was used in analysis of overall survival. The numbers of high Notch expression group and low Notch expression group are 50, respectively.

In this study we also conducted further research to investigate how the survival of GC patients was affected by Notch. Kaplan–Meier Plotter tools were applied to analyze the correlation between Notch mRNA levels and the survival of patients with GC in 882 gastric tumors. According to the results of analysis, all the sampled GC patients were negative correlated with mRNA expression of all the four types of Notch in terms of post-progression survival (PPS), progression-free survival (FP), and overall survival (OS) (p < 0.05) (Figure 2A–2D). We further validated these results through the analysis of samples from gastric adenocarcinoma patients with immunohistochemistry (Figure 1C) and survival analysis. The patients with higher Notch1/2/3/4 expression levels had worse overall survival than those with lower Notch1/2/3/4 expression levels (Figure 3A–3D). Generally, lower Notch mRNA expression levels in GC patients indicated higher PPS, FP, and OS.

### Cellular functions, pathways and frequently altered neighbor genes predicted to be affected by changes in Notch receptors in patients with GC

**Figure 4:**
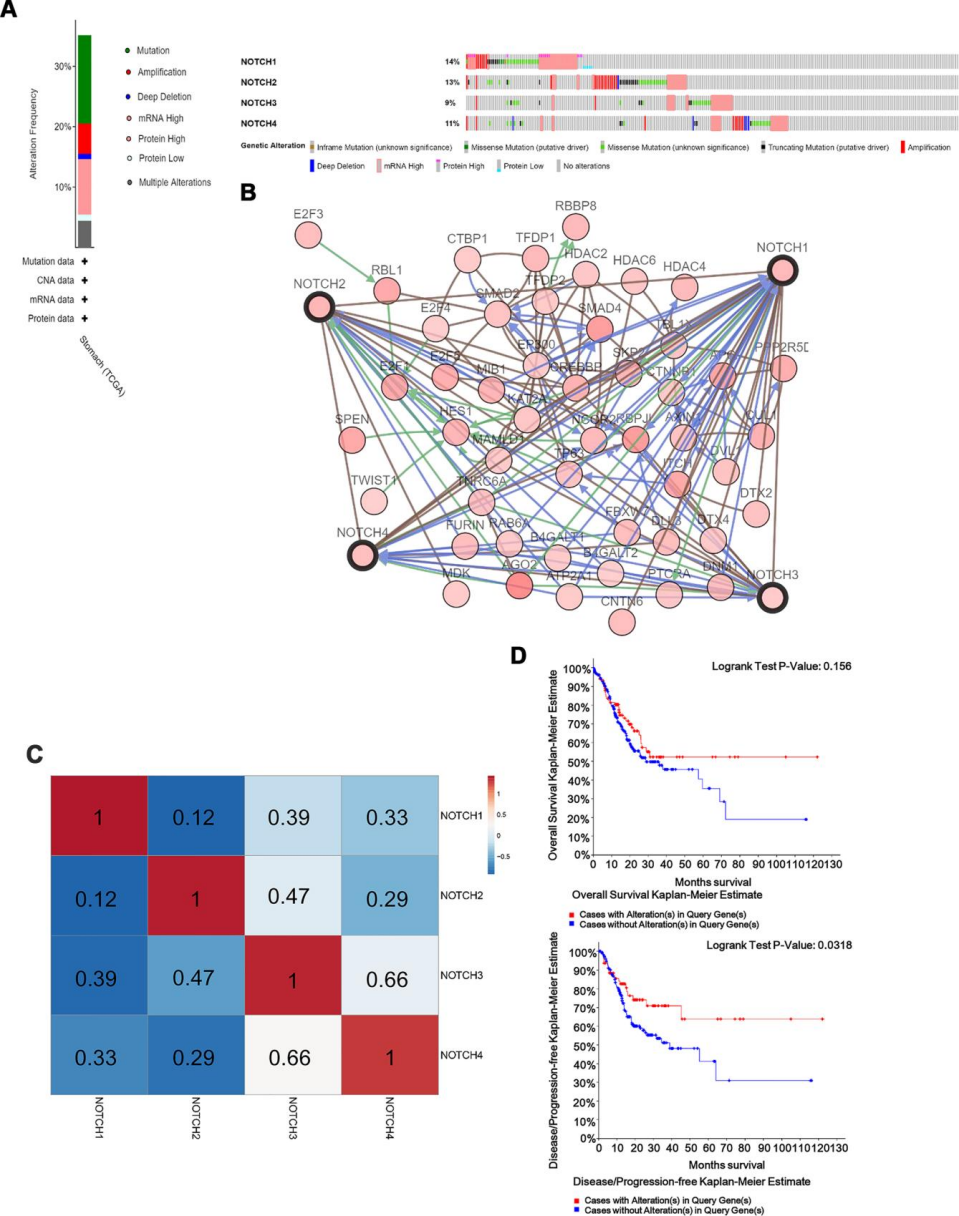
**Notch genes expression and mutation analysis in GC.** (**A**) Notch1, Notch2, Notch3 and Notch4 mutation rates were 14%, 13%, 9% and 11%, respectively. (**B**) Network for 4 Notch receptors and the 50 most frequently altered neighbor genes. (**C**) Genetic alterations in Notch receptor were correlated with longer DFS in gastric adenocarcinoma patients. (**D**) The relationship between genetic alterations in Notch and OS/DFS. Logrank test was used in analysis of OS/DFS.

In this study, the cBioPortal online tool was used for analysis of Notch alterations, correlation, and networks. Notch receptors were altered in 223 samples out of 478 patients with stomach adenocarcinoma (47%). Two or more alterations were detected in 51 samples (10%) (Figure 4A). Subsequently, a Notch network was built up using the 50 most frequently altered neighbor genes. According to the results, there was a close correlation between the alterations of Notch and cell cycle-related genes, including E2F1, E2F3, E2F4 and E2F5, as well as histone acetylation-related genes, including HDAC2, HDAC4 and HDAC6 (Figure 4B). The analysis of mRNA was used to assess the correlation between Notch receptors with Pearson’s test. Results showed the following Notch members to be significantly positively correlated: Notch1 with Notch2, Notch3, and Notch4; Notch2 with Notch1, Notch3, and Notch4; Notch3 with Notch1, Notch2 and Notch4; Notch4 with Notch1, Notch2 and Notch3 (Figure 4C). Furthermore, a Kaplan-Meier plot and log-rank test demonstrated no significant effect of Notch genetic alterations on OS (Figure 4D, p=0.156) but were associated with disease free survival (DFS) (Figure 4D, p=0.0318) of gastric adenocarcinoma patients.

Based on the analysis of Gene Ontology (GO) and Kyoto Encyclopedia of Genes and Genomes (KEGG) using the Database for Annotation, Visualization, and Integrated Discovery (DAVID), we predicted a significant correlation between Notch alterations and functional activities. The function of target host genes were predicted via GO enrichment analysis in terms of three aspects, namely, biological processes, cellular components, and molecular functions. Enrichment analysis was applied to investigate the biological functions of these target genes. According to the enriched gene ontology (GO) cell component (CC) analysis, there was significant enrichment for genes related to the Golgi membrane, endoplasmic reticulum membrane, and receptor complex (Supplementary Figure 1A). For biological processes (BP), there was an enrichment in receptor activity, calcium ion binding and enzyme binding (Supplementary Figure 1B). In addition, in terms of molecular function (MF) results were mainly associated with the Notch signaling pathway and transcription initiation from RNA polymerase II promoter (Supplementary Figure 1C). The KEGG disease terms were mainly enriched in cancers, cancers of the digestive system and musculoskeletal diseases (Supplementary Figure 1D). We also observed significant enrichment in KEGG pathways (Supplementary Figure 1E). Among these pathways, cell cycle, TGF-β signaling pathway, Notch signaling pathways, microRNA in cancer, and Wnt signaling pathway were involved in the tumorigenesis and pathogenesis of GC (Supplementary Figures 2A and 2B).

### Notch expression is correlated with immune infiltration levels in gastric cancer

**Figure 5:**
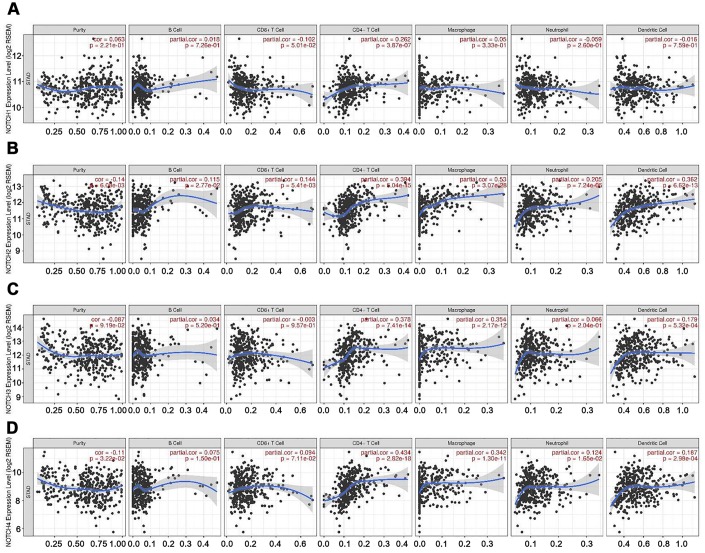
**Correlation of Notch expression with immune infiltration level in GC.** (**A**) Notch1 expression had significant positive correlations with infiltrating levels of CD4+ T cells (n = 415). (**B**) Notch2 expression had significant correlations with infiltrating levels of CD4+ T cells, macrophages, neutrophils, and dendritic cells in GC (n = 415). (**C**) Notch3 expression was significantly related to infiltrating levels of CD4+ T cells and macrophages in GC but no significant correlation with infiltrating level of B cells (n = 415). (**D**) Notch4 expression had significant positive correlations with infiltrating levels of CD4+ T cells and macrophages in GC (n = 415). Spearman’s correlation was used to analyze correlation between Notch receptors and immune cells.

Tumor-infiltrating lymphocytes have been used to predict sentinel lymph node status and survival in cancers [24]. Hence, we explored the correlation between the levels of immune infiltration and the expression of Notch in gastric adenocarcinoma patients based on TIMER. Interestingly, we found high levels of Notch mRNA expression to be associated with high immune indiltration in gastric adenocarcinoma. Notch1 mRNA expression level was significantly positively correlated with infiltrating levels of CD4+ T cells (r = 0.262, P = 3.87e-07) (Figure 5A). Notch2 mRNA expression level was significantly positively correlated with infiltrating levels of CD4+ T cells (r = 0.394, P = 5.04e-15), macrophages (r = 0.53, P = 3.07e-28), neutrophils (r = 0.205, P = 7.24e-05) and dendritic cells (DCs) (r = 0.362, P = 6.62e-13) (Figure 5B). Notch3 mRNA expression level was significantly positively correlated with infiltrating levels of CD4+ T cells (r = 0.378, P = 7.41e-14) and macrophages (r = 0.354, P = 2.17e-12) (Figure 5C). Similarly, Notch4 mRNA expression was positively correlated with infiltrating levels of CD4+ T cells (r = 0.434, P = 2.82e-18) and macrophages (r = 0.342, P = 1.30e-11) (Figure 5D). In addition, this study did not find significant correlations between the expression of Notch and infiltrating levels of B cells and CD8+ T cells in gastric adenocarcinoma. These findings strongly indicated that Notch played an important role in immune infiltration in gastric adenocarcinoma, particularly for CD4+ T cells and macrophages.

### Correlation analysis between Notch expression and immune markers

In order to detect the relationship between Notch and the diverse immune infiltrating cells, we concentrated particularly on the correlations between Notch and immune markers of various immune cells in gastric adenocarcinoma using the TIMER databases. We analyzed different immune cells including tumor-associated macrophages (TAMs), neutrophils and DCs in gastric adenocarcinoma (Table 2). Meanwhile, Th1 cells, Th2 cells and Tregs were also analyzed. After adjusting the correlation for tumor purity, results demonstrated that the Notch mRNA expression level had significant correlations with TAMs, DCs, CD4+ T cells and neutrophils in gastric adenocarcinoma (Table 2). Specifically, we showed chemokine (C-C motif) ligand (CCL)-2, IL-10, CD163, CSF1R and FCGR2A of TAMs, ITGAX, CD1C, NRP1 and THBD of DCs, CCR7, ITGAM of neutrophils, STAT4 of Th1 cells, GATA3, CXCR4, CCR4 and CCR8 of Th2 cells, STAT5B and TGFB1 of Tregs to be significantly correlated with the expression of Notch2 in gastric adenocarcinoma (Table 2). High Notch3/4 expression was associated with high levels of infiltration of macrophage and CD4+ T cells in gastric adenocarcinoma. TAM markers such as CCL2 and CSF1R, DCs markers such as ITGAX, NRP1 and THBD, neutrophils markers such as CCR7 and ITGAM, Th2 markers such as GATA3, CXCR4, CCR4 and CCR8 and Treg markers such as STAT5B and TGFB1 showed significant correlation with Notch3 expression. TAM markers such as CCL2 and CSF1R, DCs markers such as ITGAX, CD1C, NRP1 and THBD, neutrophils markers such as CCR7 and ITGAM, Th2 markers such as GATA3, CXCR4, CCR4, Treg markers such as FOXP3, STAT5B, TGFB1 were also significantly correlated the expression of Notch4. Based on all the findings above, we provide evidence that infiltration of TAMs/Th2/Tregs is increased by expression of Notch3/4. Additionally, Notch1 had a positive correlation with CD4, GATA3 and STAT5B and a negative correlation with CD59 in gastric adenocarcinoma. Overall, these results further confirm the critical role of Notch in inhibiting immune activities in the gastric adenocarcinoma microenvironment.

**Table 2:** Correlation analysis between Notch and related markers of immune cells.

**Description**	**Gene marker**	**NOTCH1**		**NOTCH2**		**NOTCH3**		**NOTCH4**	
		**Cor**	**P**	**Cor**	**P**	**Cor**	**P**	**Cor**	**P**
TAM	CCL2	0.052	0.313	0.264	***	0.239	***	0.27	***
	IL10	0.081	0.117	0.27	***	0.171	***	0.199	***
	CD163	0.097	0.058	0.454	***	0.193	***	0.163	**
	VSIG4	0.019	0.711	0.402	***	0.177	***	0.099	0.053
	CSF1R	0.066	0.198	0.466	***	0.251	***	0.235	***
	FCGR2A	-0.012	0.22	0.337	***	0.168	**	0.125	*
	FCER2	0.141	**	0.249	***	0.197	***	0.327	***
DCs	ITGAX	0.111	*	0.327	***	0.252	***	0.278	***
	CD1C	0.031	0.55	0.396	***	0.182	***	0.287	***
	NRP1	0.135	**	0.483	***	0.461	***	0.507	***
	THBD	0.164	**	0.453	***	0.518	***	0.598	***
Neutrophils	CCR7	0.15	**	0.324	***	0.209	***	0.338	***
	ITGAM	0.105	*	0.479	***	0.286	***	0.28	***
	CD59	-0.323	***	0.311	***	0.152	**	0.153	**
Th1	STAT4	0.047	0.363	0.316	***	0.075	0.146	0.152	**
	TBX21	0.149	**	0.185	***	0.088	0.088	0.148	**
	CD4	0.324	***	0.178	***	0.324	***	0.197	***
Th2	GATA3	0.22	***	0.173	***	0.22	***	0.2	***
	CXCR4	0.097	0.059	0.375	***	0.213	***	0.288	***
	CCR4	0.169	***	0.408	***	0.24	***	0.3	***
	CCR8	0.137	**	0.347	***	0.216	***	0.198	***
Treg	FOXP3	0.136	**	0.173	***	0.176	***	0.204	***
	STAT5B	0.351	***	0.584	***	0.399	***	0.414	***
	TGFB1	0.152	**	0.373	***	0.532	***	0.384	***

*P<0.05, **P<0.01, ***P<0.001

## DISCUSSION

The importance and effect of the Notch signaling pathway on cellular activities and in cancer with either oncogenic or tumor-suppressive functions have been widely recognized [2, 25, 26]. Notch signaling functions as juxtacrine signaling between cells. This type of signaling allows Notch to regulate heterotypic interactions between stroma and tumor. These interactions are known to be of importance in various aspects of tumor biology, such as angiogenesis, cancer stem cell maintenance, immune infiltration, and resistance to therapy. Despite the already confirmed effects of Notch signaling on GC, the functions of different Notch family members in GC remain to be elucidated. In this study, we conducted detailed analyses on various members of Notch referring to mutation, expression and prognostic values in GC patients.

Results from our study showed that over-expression of mRNA and protein were found in Notch family members. Also, higher mRNA expressions of Notch1/2/3/4 were significantly associated with shorter OS in GC patients. Moreover, a high Notch mutation rate (47%) was observed in gastric adenocarcinoma patients. Further, genetic alteration of Notch receptors was associated with shorter DFS in gastric adenocarcinoma patients. Finally, the functions and pathways affected by mutations in Notch receptors as well as the most significant 50 frequently altered neighbor genes in gastric adenocarcinoma patients were analyzed. Results showed that cell cycle-related genes, including E2F1, E2F3, E2F4 and E2F5, and histone acetylation-related genes, including HDAC2, HDAC4 and HDAC6 were significantly affected by mutations in Notch receptors. Results also showed the following to be remarkably regulated by Notch mutation in GC: biological processes such as receptor activity, calcium ion binding and enzyme binding; cellular components such as Golgi membrane, endoplasmic reticulum membrane, and receptor complex; molecular functions such as Notch signaling pathway and transcription initiation from RNA polymerase II promoter; KEGG disease terms such as cancers, cancers of the digestive system and musculoskeletal diseases; KEGG pathways such as cell cycle, Notch signaling pathways and TGF-β signaling pathway.

Of note, Notch1 played a critical role in regulating the senescence secretome in fibroblasts [27], which was perhaps a part of its function in the regulation of stromal activation in the process of tumorigenesis. In breast cancer models, Notch1 was induced by fibroblast-derived CCL2 to maintain a stem cell phenotype and had a possible oncogenic role [17, 28]. Importantly, Notch1 activation enabled primary melanoma cells to acquire metastatic capabilities [29]. In gastric cancer, the AKT1/NF-kB/Notch1/PTEN axis had a significant role in the development of chemoresistance [30]. Notch1 activation also showed correlation with GC progression and was defined as an independent prognostic factor [31]. In B cell lymphoma, Jag1 induced the expression of FGF4 which in turn activated Notch2 in lymphoma cells [32]. Notch ligand Jag1 interacted with Notch3 to regulate the resistance [33]. Moreover, Notch3 triggered by stromal cell-derived exosomes activated antiviral signaling depending on STAT1 in cancer cells [33]. Induction of Notch3 by CAFs led to an increase in proliferation of cancer stem cells [34]. Meanwhile, the renewal of cancer stem cell could be induced by IL-6 through Notch3 in breast cancer [35]. Notch4 had a causative role in the tumorigenesis [36] and might affect the development of fibroblasts [37]. Recent data also showed that Notch is involved in liver glucose and lipid homeostasis [38, 39].

Another important aspect of this study was that Notch expression was correlated with diverse immune infiltration levels in gastric adenocarcinoma. Notch1 mRNA expression level was significantly positively correlated with infiltrating levels of CD4+ T cells. Notch2 mRNA expression level was significantly positively correlated with infiltrating levels of CD4+ T cells, macrophages, neutrophils and DCs. Notch3 mRNA expression level was significantly positively correlated with infiltrating levels of CD4+ T cells and macrophages. Moreover, the correlation between Notch expression and the marker genes of immune cells imply the role of Notch in regulating tumor immunology in gastric adenocarcinoma. The M2 macrophage markers such as CD163, VSIG4, and CSF1R showed correlations with Notch2 expression. These results indicated the potential role of Notch2 in regulation of TAMs polarization. Furthermore, our results revealed that Notch had the potential to activate Tregs. The increase in Notch expression positively correlates with the expression of Treg markers (FOXP3, CCR8, STAT5B in Table 2). In addition, there were significant correlations between Notch expression and several markers of T helper cells (Th1, Th2), DCs and neutrophils in gastric adenocarcinoma. These correlations could be indicative of a potential mechanism that Notch regulated immune cells in gastric adenocarcinoma. Together these findings suggested that Notch play a significant role in recruitment and regulation of immune infiltrating cells in gastric adenocarcinoma.

In this study, a systemic analysis was performed on Notch receptors expression, mutation and GC patients’ prognosis, which provided a further understanding of the biomolecular properties of GC. Our results demonstrated that the high expression of Notch1/2/3/4 in GC tissues might exert a significant function in GC tumorigenesis. High Notch1/2/3/4 expression could also act as molecular markers to categorized high-risk subgroups of GC patients. According to the present study, Notch1/2/3/4 could be potential therapeutic targets for GC, and transcription levels of Notch1/2/3/4 could be potential prognostic markers overall promoting GC survival and prognostic accuracy.

## MATERIALS AND METHODS

### ONCOMINE analysis

Using the online cancer microarray database, ONCOMINE (https://www.oncomine.org/), we analyzed the transcription levels of Notch in various cancers tissues. A students’ t-test was performed to conduct a comparative analysis of the different Notch expression in normal tissues versus cancer tissue samples. The cut-off value for a significant fold change was 1.5, while the cut-off value for significant p value was 0.0001.

### GEPIA dataset

GEPIA (http://gepia.cancer-pku.cn/), a web server for analyzing the sequencing expression data of RNA based on 9,736 tumors and 8,587 normal samples from the cancer genome atlas (TCGA) and the GTEx projects [40], was used for analysis of differential expression.

### The Kaplan-Meier plotter

Kaplan-Meier Plotter (https://kmplot.com/) was applied to the assessment the prognostic value of Notch. This online database provides gene expression data and information on patients’ survival performance based on 882 clinical GC cases [41]. All the sampled patients were categorized into groups based on median expression of Notch (high vs. low expression) to analyze the overall survival (OS), progression-free survival (FP), and post progression survival (PPS) of GC patients using a Kaplan-Meier survival plot. We only chose the best JetSet probe set of Notch to obtain Kaplan-Meier plots.

### TCGA data and cBioPortal

TCGA provides sequencing and pathology data for over 30 different cancers [42]. We used the dataset for stomach adenocarcinoma, which included 478 cases with pathology reports to further analyze Notch expression using cBioPortal (https://www.cbioportal.org/) [43]. From the genomic profiles, we obtained data regarding protein expression Z-scores (RPPA), mRNA expression z-scores, putative copy-number alterations (CNA) from GISTIC and mutations. Co-expression and network analysis were conducted following online instructions of cBioPortal.

### Immunohistochemistry

Tissue samples were fixed in 4% paraformaldehyde and embedded in paraffin. Anti-Notch1 (1:200; CST, Cat#: 3608, Danvers, MA, USA), anti-Notch2 (1:200; CST, Cat#: 5732), anti-Notch3 (1:200; Abcam, Cat#: ab23426, Cambridge, MA, USA) and anti-Notch4 (1:200; Abcam, Cat#: ab222400) were used as primary antibodies. Immunohistochemistry was performed according to the method previously described [44–46]. Gastric cancer tissues and adjacent normal gastric tissues were obtained from the Huashan Hospital (Shanghai, China).

### Immune infiltrates analysis in TIMER database

TIMER is a comprehensive tool established for analyzing immune infiltrates across different types of cancer (https://cistrome.shinyapps.io/timer/) [47]. We analyzed Notch expression in different cancer types and the correlation of Notch expression with the abundance of immune infiltrates. Meanwhile, correlations between Notch expression and gene markers of tumor-infiltrating immune cells were also explored.

## Supplementary Material

Supplementary Figures
