# Supplementary material for: Notch1/2/3/4 are prognostic biomarker and correlated with immune infiltrates in gastric cancer
Source: Aging (Albany NY). 2020 Feb 6;12(3):2595–609. doi: 10.18632/aging.102764 (PMC7041744; doi:10.18632/aging.102764)
Supplement: Supplementary Figures [file aging-12-102764-s001..pdf]

SUPPLEMENTARY FIGURES

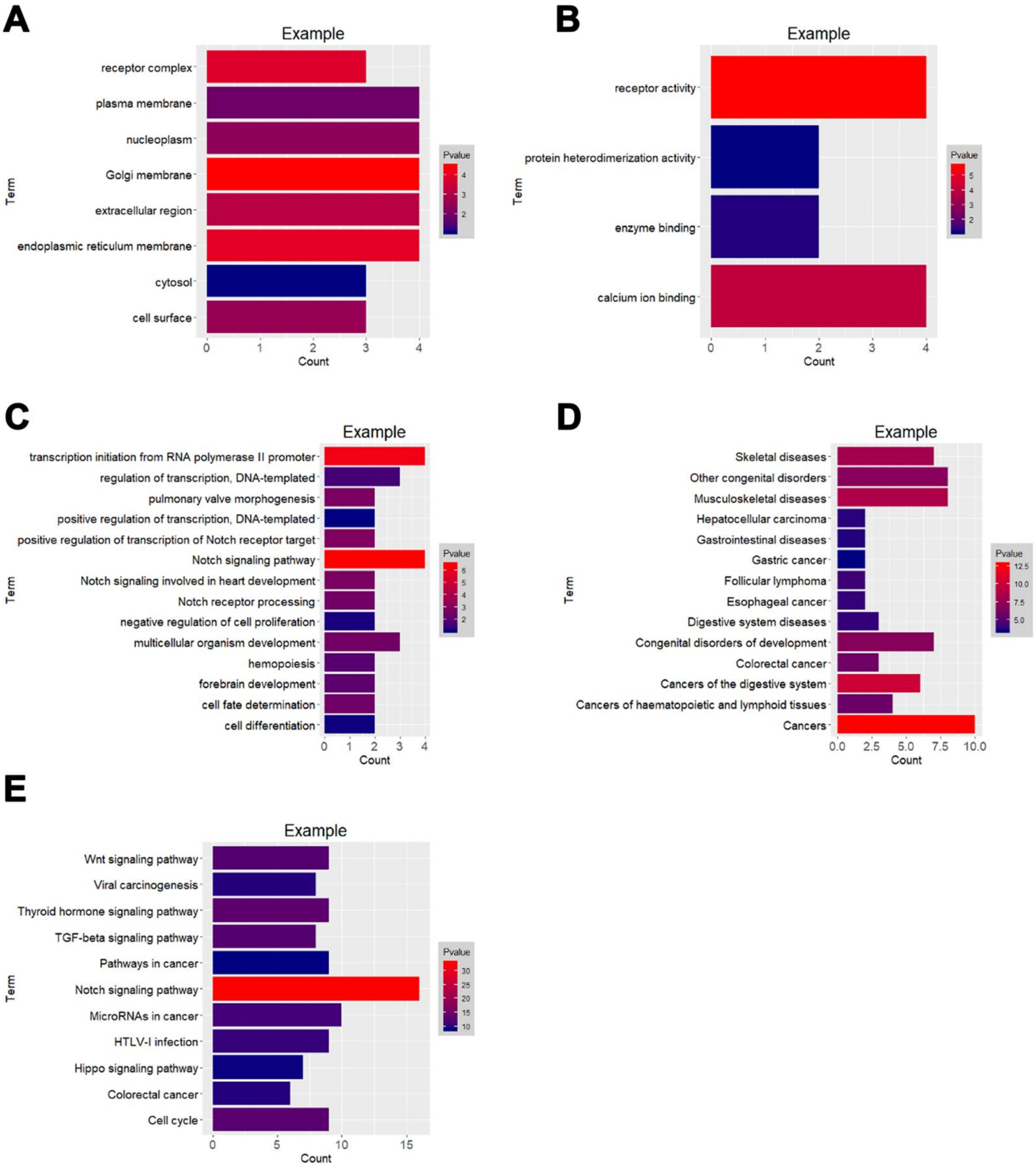

Supplementary Figure 1. The functions of Notch and genes significant associated with Notch alterations were predicted. (**A**) Cellular components. (**B**) Biological processes. (**C**) Molecular functions. (**D**) KEGG disease. (**E**) KEGG pathway.

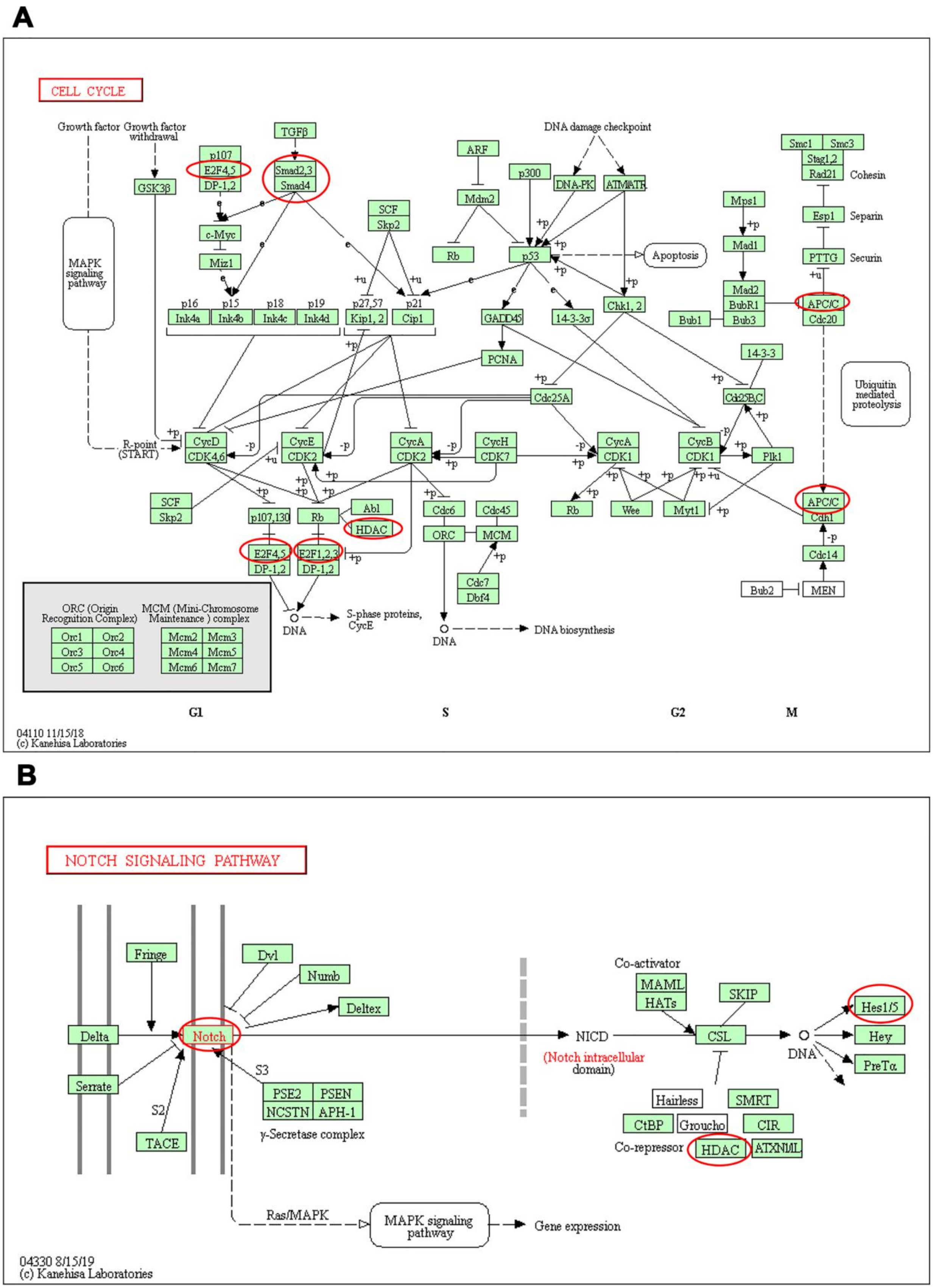

Supplementary Figure 2. Cell cycle and Notch signaling pathway regulated by the Notch alteration in GC. The (**A**) cell cycle and (**B**) Notch signaling pathway regulated by the Notch alteration in GC (cBioPortal) are shown.
